# Synthesis and Characterizations of Eco-Friendly Organosolv Lignin-Based Polyurethane Coating Films for the Coating Industry

**DOI:** 10.3390/polym14030416

**Published:** 2022-01-20

**Authors:** Sara Bergamasco, Swati Tamantini, Florian Zikeli, Vittorio Vinciguerra, Giuseppe Scarascia Mugnozza, Manuela Romagnoli

**Affiliations:** Department of Innovation in Biological, Agro-Food and Forest Systems (DIBAF), University of Tuscia, 01100 Viterbo, Italy; sara.bergamasco@unitus.it (S.B.); vincigue@unitus.it (V.V.); gscaras@unitus.it (G.S.M.)

**Keywords:** wood coating, wood color, contact angle, SEM, Py-GC/MS, FTIR mapping, beech, bio-circular economy

## Abstract

Three different formulations of bio-based polyurethane (PU), varying the weight ratio between Organosolv lignin and a commercial isocyanate, were synthesized. The coating formulations were characterized by SEM, pyrolysis-GC/MS, FTIR spectroscopy and FTIR mapping, which confirmed the successful formation of urethane bonds between commercial isocyanate and hydroxyl groups deriving from lignin. The coatings were applied on beech wood samples to measure color and contact angles, and eventually FTIR mapping of the coated wood samples was performed. FTIR mapping is an interesting tool to monitor the distribution of PU chemical bonds on the coating surface and to evaluate the homogeneity of the applied coating films. Increasing the lignin content of the PU coatings results in more red-yellow and darker tones, while the commercial PU coating is transparent. For a higher lignin concentration, the solid content as well as the weight gain of the applied coatings increase. A higher percentage of lignin in the prepared PU formulations leads to superficial cracks and therefore higher coating permeability compared to the commercial PU, but the prepared lignin-based PU coating still makes a raw wood surface significantly more hydrophobic. Apparently, additives such as film-formers with low surface tension to counteract cracks’ formation are necessary to improve the performance of lignin-based PU coatings.

## 1. Introduction

Wood materials and wood-based products are increasingly considered the materials of the future, fully in line with modern concepts of a bio-circular economy [1,2]. However, the intrinsic biodegradability of wood is the most important weakness, which limits a wider end use, and thus the improvement of wood durability is still one of the major challenges [3,4]. This challenge can be tackled by different means, such as thermo-treatments [5,6] or fossil-based and natural wood coatings with protective or biocidal functions [7,8,9,10].

A definition of “coating material” is given by the European Standard EN 971-1 [11]: “A product, in liquid, in paste or powder form, that, when applied to a substrate, forms a film possessing protective, decorative and/or other specific properties”. Various types of organic substances have been used in the preparation of coating materials for wood. In ancient times, only natural products such as oils or resins were available [12,13]. Later on, the application of thermal or chemical modifications improved the performance of natural products. A continuously developing chemical industry in the first half of the 20th century produced a wide range of synthetic coating films, such as alkyds, polyurethanes and polyesters, which were successfully applied in many fields, including wood technology [14,15,16,17,18]. For the modern coating industry, polyurethanes (PUs) present an important class of polymers due to outstanding mechanical, chemical and physical properties. Thus, they find application in many industrial sectors in the form of flexible or rigid foams, coatings, adhesives, elastomers, thermoplasts or thermosets [8,19]. Modern PU coating applications include self-healing coating films that can also be applied to rather rough surfaces, such as wood [6]. Polyurethanes are available as solvent or water-borne formulations, and polymer curing may be brought about at ambient or elevated temperatures. One of the major issues of polyurethanes is their fossil-based origin and the toxicity of reactants and pre-cursors such as isocyanates, which act as a modifier or crosslinking agent, rather than the main film former. Conventional PUs are usually synthesized by a polyaddition reaction between polyols and poly-isocyanates. These two, when reacted together, form the urethane linkages observed in a crosslinked polymer. Recently, several attempts were reported to substitute fossil-based building blocks of PU coatings with bio-based compounds because the versatility in chemistry and processing enables wide opportunities for introducing new raw materials into various PU products [20]. Recent research utilizes castor oil for the preparation of a hyperbranched polyol that was further used for a bio-based PU coating with self-cleaning abilities [21]. Besides vegetable oils (i.e., from soybean) or starch, which have been extensively applied as natural and renewable polyol sources for PUs, lignin has emerged as a promising alternative polyol source for application in resins, adhesives and foams [22,23]. After cellulose, lignin is the second most abundant natural polymer, containing a broad range and high contents of chemical functional groups such as hydroxyls, carbonyls and carboxyls [24], which allow for its utilization as an alternative for polymer development, especially as a substitute for petroleum-based polyols in polyurethane systems. Scientific contributions regarding the substitution of polyols by lignin in polyurethanes involve, in general, kraft lignins [25], while the performance of other technical lignins, i.e., Organosolv lignins, has not yet been evaluated. Thus, the objective of this article is to examine the use of technical Organosolv lignin, a waste stream from the pulping industry, as a polyol substitute to produce bio-based polyurethane coatings, which eventually provide a more sustainable alternative with less risk for human and environmental health by reducing the content of synthetic and fossil-based components. The lignin derives from species of short supply chain, which is one of the main topics of a bio-circular economy where alternative uses of wood waste in the substitution of synthetic compounds in coating or adhesive formulations are envisioned [1,26].

## 2. Materials and Methods

### 2.1. Materials

Flat-grained beech (*Fagus sylvatica* L.) wood samples, 15 mm × 15 mm × 3 mm (L × T × R), were taken in tangential direction from one cut board. Wood samples were conditioned in the lab at 65% relative humidity (RH) and 21 °C for one year. The commercial PU coating provided by the company Finedin S.r.l. (Taviano, Italy) is a PU-based synthetic flattener for wood.

Hardwood (Beech) Organosolv lignin (OL) was supplied by Fraunhofer Italia S.c.r.l. (Bolzano, Italy). Desmodur^®^L75 is an aromatic polyisocyanate containing an isomeric mix of toluene diisocyanate (TDI), diethylene glycol and trimethylolpropane, and it was supplied by Covestro LLC (Leverkusen, Germany). 2-Methyltetrahydrofuran (2-MeTHF) was obtained from Carlo Erba Reagents (Cornaredo, Italy).

The different formulations and relative abbreviations used in the text are explained in Table 1.

### 2.2. Methods

#### 2.2.1. Polyurethane Synthesis

Three formulations of bio-based polyurethane, varying the weight ratio between lignin and isocyanate, were synthesized and eventually named as PU-3:1, PU-1:1 and PU-1:3. The lignin was dispersed in 5 mL of 2-MeTHF (2-Methyltetrahydrofuran) with a dispersion degree equal to 20%, 40% and 60% (wt/vol) and stirred magnetically for 30 min at room temperature. After that, Desmodur^®^L75 equal to 60%, 40% and 20% (wt/vol) was added to the lignin solutions. For a complete dissolution of isocyanate and eventual polyurethane formation, the solutions were left under magnetic stirring for an additional 60 min at room temperature. Subsequently, the PU formulations were applied as coatings on the wood samples. Aliquots of the three formulations were poured in aluminum plates for subsequent heat-induced finalization of the crosslinking reaction in a drying oven (60 °C, 72 h) and eventual solid content determination (see below). The NCO/OH molar ratio was calculated based on the equation below [27]:

(1) $$\frac{\mathrm{NCO}}{\mathrm{OH}}=\frac{W_{\mathrm{TDI}}{\left[\mathrm{NCO}\right]}_{\mathrm{TDI}}}{W_{\mathrm{OL}}{\left[\mathrm{OH}\right]}_{\mathrm{OL}}}$$

where W_TDI_ is the weight (g) of isocyanate contained in Desmodur^®^L75 and W_OL_ is the weight of lignin, respectively. [NCO]_TDI_ is the molar content of isocyanate groups in TDI and [OH]_OL_ is the molar content of the total hydroxyl groups (phenolic, aliphatic and carboxylic) in lignin. For the determination of [NCO]_TDI_, the real aromatic polyisocyanate composition present in the Desmodur^®^L75 was considered. In this way, it was possible to calculate the molar ratio starting from the weight ratio. Molar ratios equal to 1.96, 0.65 and 0.21 were obtained for the formulations PU-3:1, PU-1:1 and PU-1:3, respectively.

#### 2.2.2. Coating Application

Coatings were applied onto one side of the beech wood samples using a brush (size 8) with synthetic bristles. Three layers of varnish were applied for each sample with a drying time of 1 h between each application.

#### 2.2.3. Pyrolysis-GC/MS

About 1.5 mg of the sample PU-3:1 was pressed in a special syringe and directly pyrolyzed at 450 °C in a Pyrojector II (SGE Analytical Science, Trajan Scientific and Medical, Melbourne, Australia). The pyrolysis chamber was maintained at 450 °C. Pyrolysis products were separated in a GCMS-QP5050 instrument (Shimadzu Corporation, Kyoto, Japan). Helium was the carrier gas at a pressure of 100 kPa in the pyrolyzer and 70 kPa in the GC injector (280 °C, 1–20 split ratio). The initial temperature in the oven was 45 °C for 4 min, then increased to 240 °C at a rate of 4 °C min^−1^ and finally until 280 °C at a rate of 39 °C min^−1^. Pyrolysis products were identified by mass spectra interpretation and comparison with NIST and Wiley computer libraries and reference literature [28,29]. For each pyrogram, normalized at the most intense peak, the relative peak areas of 26 principal phenolic lignin pyrolysis products as well as the respective PU pyrolysis products were determined.

#### 2.2.4. SEM

One representative sample (PU-3:1) was used for a morphological characterization of the coating by Scanning Electron Microscope (SEM) analysis. The sample was attached to aluminum stubs by carbon tape and sputter-coated with gold in a Balzers MED 010 unit (Oerlikon Balzers, Balzers, Liechtenstein). A JEOL JSM 6010LA SEM was used (JEOL Limited, Tokyo, Japan). The sample to be analyzed was selected after observation on a FTIR microscope.

#### 2.2.5. Weight Gain and Coating Film Thickness

The weight of 12 samples for each formulation was measured before and after coating application using the electronic analytical balance JA503 (Fulltech Instruments S.r.l., Rome, Italy). The second weight was taken after one week of drying (168 h). The percentage of weight gain was calculated using Equation (2) below:

(2) $$W_{\mathrm{gain}}=\frac{W_{2}-W_{1}}{W_{1}}\;\cdot \;100$$

where W_gain_ is the weight gain of the wooden sample after coating application, W_1_ is the weight of uncoated samples and W_2_ is the weight of coated samples.

For determination of coating thickness, three samples per coating were halved and the coating layer was measured in five spots/sample using a stereomicroscope (model MZ 16A, Leica Microsystems AG, Wetzlar, Germany).

#### 2.2.6. Solid Content Analysis

Samples of fresh varnishes were weighed using the electronic analytical balance JA503 (Fulltech Instruments S.r.l., Rome, Italy) and left in the oven (FD 115, Binder GmbH, Tuttlingen, Germany) at 60 °C for 72 h. After drying, the samples were weighed again and solid content (SC) in percentage was calculated using Equation (3):

(3) $$\mathrm{SC}=\frac{W_{\mathrm{dry}}}{W_{\mathrm{fresh}}}\;\cdot \;100$$

where SC is the solid content, W_dry_ is the weight after the drying period and W_fresh_ is the weight of fresh varnish before oven-drying.

#### 2.2.7. Color Measurements

Digital images of the samples were taken using an HP Scanjet 4800 scanner, in order to analyze their CIELAB coordinates using the open-source graphics software GNU Image Manipulation Program (GIMP v2.10.12). According to UNI EN ISO/CIE-11664-4:2019 [30] and UNI EN 927-6:2019 [31], five different spots per sample were selected and three samples per coating formulation were analyzed. The coordinates L*, a* and b* were measured on the tangential plane. The total color difference (ΔE*) represents the difference in color between virgin wood and the samples coated with PU formulations and the commercial PU coating. ΔE* was calculated according to Equation (4):

(4) $$\Delta E^{*}=\sqrt{{\left(L_{2}^{*}-L_{1}^{*}\right)}^{2}+{\left(a_{2}^{*}-a_{1}^{*}\right)}^{2}+{\left(b_{2}^{*}-b_{1}^{*}\right)}^{2}}$$

where L* is the lightness (0 to 100), a* is the coordinate for red/green values (−120 to 120), b* is the coordinate for blue/yellow values (−120 to 120), Index 1 stands for the reference sample and Index 2 stands for the sample to analyze.

#### 2.2.8. Optical Contact Angle

Optical contact angle (CA) measurements on the PU film were performed at room temperature using the optical tensiometer Attension Theta Flex (Biolin Scientific Group, Gothenburg, Sweden). Three samples per treatment were chosen, and two spots per specimen were analyzed. A droplet of deionized water (5 µL) was released on the three PU formulations, on the commercial PU and on three control samples. CA measurements after water drop release (CA-T0), after 10 s (CA-T10) and after 600 s (CA-T600) of drop deposition were recorded. Data elaborations were conducted using the software OneAttension v4.1.2 (r9576), provided by Biolin Scientific Group (Gothenburg, Sweden).

#### 2.2.9. FTIR Spectroscopy and Mapping

After oven-drying at 60 °C for 72 h (FD 115, Binder GmbH, Tuttlingen, Germany), potassium bromide (KBr) pellets (Ø = 7 mm) were prepared for each coating formulation with a sample concentration of 2 wt.% using a Specac mini-pellets press at 2 bar for 5 min (Specac Inc., Orpington, UK). FTIR spectra were recorded in absorbance mode in the range of 4000–400 cm^−1^ with a FTIR-4100 Fourier Transform Infrared spectrometer (Jasco Corp., Easton, MD, USA). All FTIR spectra were smoothed, baseline-corrected and normalized for the most intense peak of the spectra using Spectra Manager software v2.15.01 (Jasco Corp., Easton, MD, USA). One representative wood sample per coating formulation was analyzed using a Jasco IRT-7000 Irtron Infrared microscope (Jasco Corp., Easton, MD, USA) in order to investigate the surface of the coated samples using FTIR imaging. The number of scans was 500 per measurement point and the analyzed area was 270 μm × 270 μm.

#### 2.2.10. Statistical Analysis

After verifying the normal distribution of data with the Anderson–Darling method, the differences among formulations were investigated by ANOVA (Minitab^®^ v18.1, Minitab Inc., State College, PA, USA). ANOVA was carried out on color change, weight gain and contact angle measurements. The statistical analysis considered a 95% confidence interval (CI). If significant differences were detected in the ANOVA (F test with *p* ≤ 0.05), the Tukey test (*p* ≤ 0.05) was applied to compare the series.

## 3. Results and Discussion

**Scheme 1:**
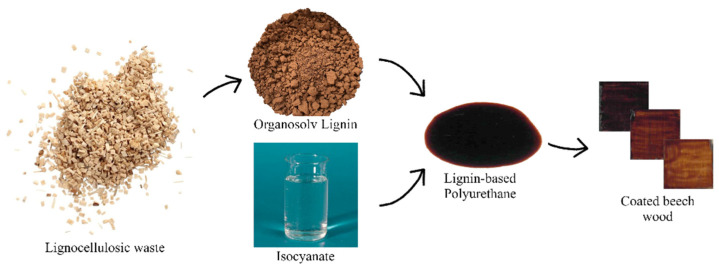
Overview of the general procedure for the preparation of the Organosolv lignin-based PU coating.

Commercial coating is transparent, soft to touch and has a shiny appearance. New formulations, with lignin-based PU, resulted shiny, but neither smooth nor transparent. The general procedure of the presented research is illustrated in Scheme 1.

Macroscopic aspects of the prepared PU coatings, such as color, weight gain, coating film thickness and contact angle, are presented first, followed by microscopic investigations of the coating surface and finally chemical analysis of the prepared PU by FTIR spectroscopy and Py-GC/MS, respectively.

### 3.1. Color Measurements

**Figure 1:**
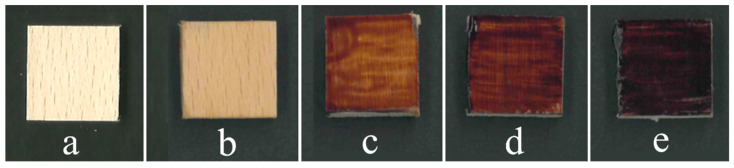
Beech wood samples before (**a**) and after coating application (**b**–**e**). (**a**) W-R, (**b**) WPU-R, (**c**) WPU-3:1, (**d**) WPU-1:1 and (**e**) WPU-1:3.

Figure 1 shows the beech wood samples coated with the different PU formulations, and their respective color coordinates are listed in Table 2.

Virgin wood (W-R) color coordinates are quite homogeneous, but after coating application, the color strongly changes. Commercial coating (WPU-R) is transparent, so the color variations compared to raw solid wood are the lowest among the other PU formulations, and wood structural elements such as rays can still be identified (Figure 1a,b). In all cases, L* is lower, which means a darker color and less transparent coating, as seen in Figure 1c–e. The coordinate a* increases for all coatings, meaning a redder color than virgin wood. For the CIELAB coordinate b*, the situation is different. In WPU-R and WPU-3:1, b* increases, rendering the color more yellow, while in WPU-1:1 and WPU-1:3, b* changes the color to a bluer tone. This could be explained by the fact that in the first two samples (Figure 1a,b), wood underneath the coating is still visible, while the last two provide a less transparent coating (Figure 1c,d), where lignin covers the surface the most. Additionally, Jusic et al. [7] and Klein et al. [32] found a gradual color change at increasing lignin content. Changing color due to all formulations is always statistically significant (ΔE*).

### 3.2. Weight, Coating Film Thickness and Solid Content

In Table 3, the measured sample weights and their respective weight gains are reported as well as the determined solid contents (SC). Weight gain was much lower for the prepared PU coatings compared to the commercial reference (WPU-R), and the difference is statistically significant. The decrease of weight gain in increasing lignin concentration is consistent with the low values of SC for the prepared PU formulations, which could be explained by the presence of other additives in the commercial products increasing its SC. Regarding the prepared formulations, an increasing lignin content led to higher SC. The coating thicknesses of the three prepared formulations are quite similar (Table 3). It is noteworthy that WPU-R shows a very high standard deviation, which might be due to higher viscosity and the presence of additives, as can be deduced by the SC.

### 3.3. Optical Contact Angle

**Figure 2:**
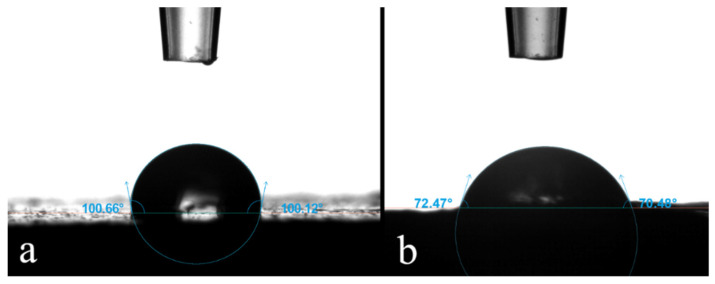
Two examples of CA measurement after drop release. In (**a**) a WPU-R sample, with a high CA, and in (**b**) a WPU-1:3 sample, with a low CA. Blue angles indicate how the instrument extrapolates the data from the images.

**Figure 3:**
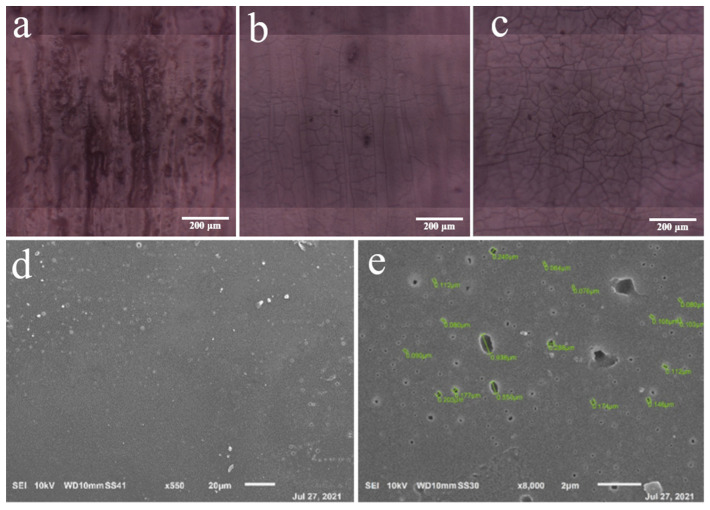
Pictures of measurement areas: (**a**) WPU-3:1, (**b**) WPU-1:1 and (**c**) WPU-1:3. SEM images of PU-3:1 (**d**,**e**).

In Table 4, the contact angle (CA) values are reported. As expected, the commercial coating had the highest CAs (Figure 2a), while higher lignin contents in the prepared PU coating led to lower CAs (Figure 2b). The CA of W-R changes strongly within the first 10 s after droplet deposition, while for WPU-R it remains stable, meaning that the water drop was adsorbed in the first case while in the latter the film is waterproof, as expected. In the coatings of the other PU formulations, the differences are not pronounced, which is why they are considered as waterproof as well, even if the CAs of the prepared PU formulations are lower than WPU-R. In the case of WPU-1:3, the water drop was completely absorbed within 10 min, as occurred with W-R. Standard deviations of prepared PU formulations are a little high, which might depend on the irregularity of the substrate, which in turn depends on the coating application. In fact, during brush application, PU formulations dried as they were applied, making the surface not flat. To summarize, it seems that the hydrophobicity of the prepared PU formulations decreased when lignin content increased. Griffini et al. [33] address this behavior to the presence of hydroxyl groups in lignin, which are higher for increasing lignin content in PU materials, leading to lower CAs. Another reason for this behavior could be explained by the cracks visible on the prepared PU surfaces (Figure 3a–c), which let the water pass through the coating film. CA values are significant only when the lignin concentration is the highest.

Figure 2 shows how the instrument extrapolates CA values by contrast between the white background and the dark droplet.

### 3.4. FTIR Microscopy and Scanning Electron Microscopy (SEM)

The pictures of the measurement zones of the FTIR mapping of the three PU formulations were captured using the multi-channel Jasco infrared microscope IRT-7000 and a Cassegrain ×16 objective. These pictures show that with the increasing lignin concentration, changes in the morphology of the material occur, causing the formation of cracks in the coating. Hence, in the image of the sample WPU-3:1, the coating surface is less cracked (Figure 3a) than WPU-1:1 (Figure 3b) and WPU-1:3 (Figure 3c). This condition was confirmed by the acquisition of scanning electron microscope (SEM) images, where a smooth and homogeneous structure (Figure 3d) was observed with the presence of a few porosities with an average diameter of 0.25 µm (Figure 3e) and a minimal presence of lignin aggregates. In contrast to other works in the literature, where significant lignin aggregation was observed using SEM [34], the PU formulations prepared in this work were rather free from lignin aggregates, as illustrated in Figure 3d,e.

### 3.5. FTIR Spectroscopy and Imaging

**Figure 4:**
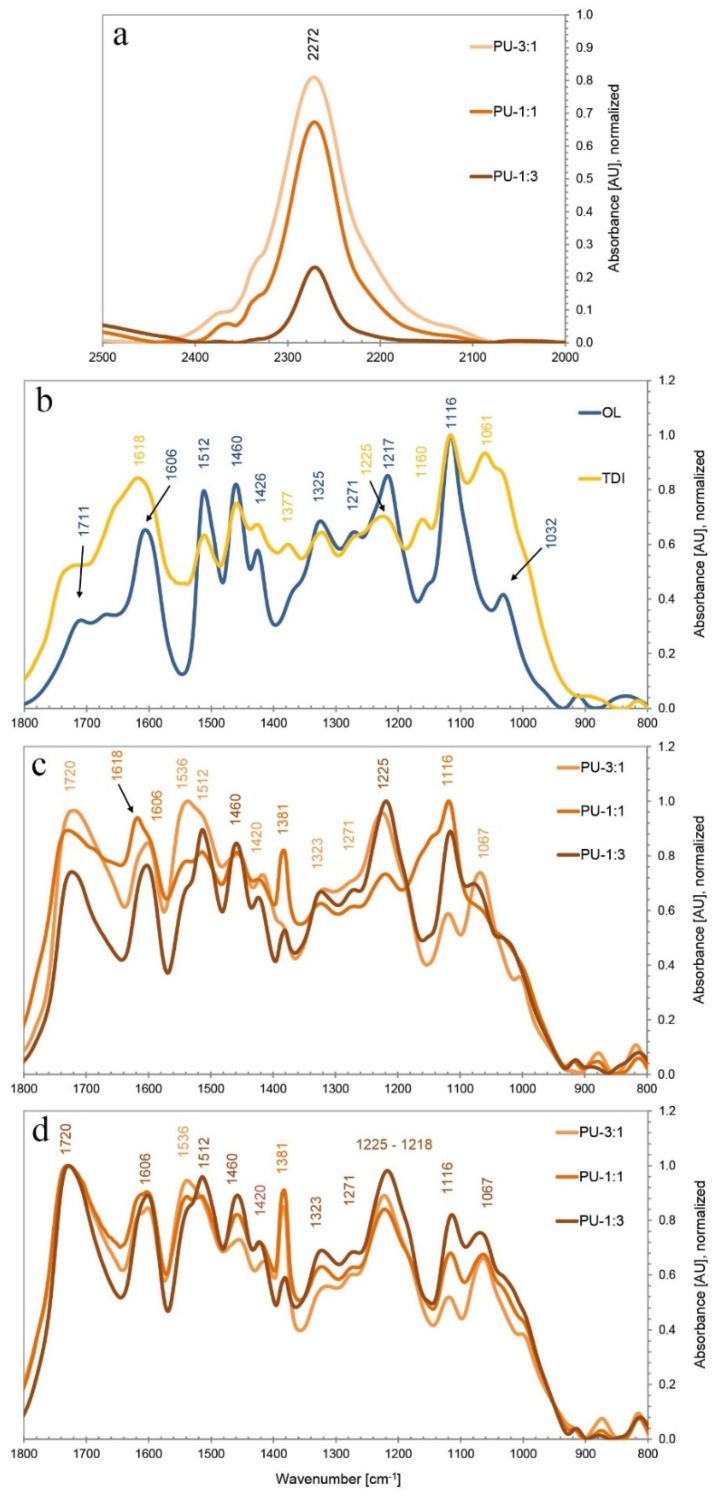
(**a**) Absorbance band of free isocyanate groups in the three different prepared PU formulations PU-3:1, PU-1:1 and PU-1:3. (**b**) FTIR spectra of Desmodur^®^L75 and Organosolv lignin (OL). The three different prepared PU formulations PU-3:1, PU-1:1 and PU-1:3 after air-drying at room temperature (**c**) and after oven-drying at 60 °C for 72 h (**d**).

In order to investigate the chemistry behind the preparation of the PU formulations, FTIR analysis was performed on samples of the three different PU formulations after both spontaneous (room temperature) and temperature-induced (60 °C for 72 h) crosslinking. The formation of urethane bonds was evaluated by studying the respective IR bands of the specific involved functional groups. The absorption band at 2270 cm^−1^ is associated to the stretching modes of the unreacted isocyanate NCO groups in the three different formulations (Figure 4a). The signal intensity of this band depends on the presence of excessive NCO groups after the formation of urethane bonds in the respective formulations. The disappearance of the isocyanate band in the sample PU-1:3 indicates a rather complete reaction between NCO and lignin OH groups, while the more intense absorption band in the spectra of the samples PU-3:1 and PU-1:1 reveals residual unreacted isocyanate in the respective formulations.

Figure 4b–d show the FTIR spectra of the three crosslinked PU formulations at room temperature (Figure 4c), and the respective FTIR spectra of the crosslinked samples after post-curing under controlled conditions (60 °C for 72 h, Figure 4d). The strong absorption band at 1720 cm^−1^ is attributable to the C=O group in the formed urethane linkages. Comparing the trends of the three different formulations, it was observed that the band is strongest in the formulation PU-3:1, followed by the formulations PU-1:1 and PU-1:3, respectively, in the case of crosslinking at room temperature. On the other hand, after crosslinking under controlled conditions, it is possible to note that the C=O band in all three different formulations increases and becomes the maximum in all three respective FTIR spectra. This indicates that the more hydroxyl groups were available with higher lignin concentration, the bigger the need for a temperature-induced crosslinking for the completion of urethane bonds’ formation. In the range 1536–1512 cm^−1^, similar trends can be found between the three formulations, with an increase in absorption in the case of crosslinking conducted under controlled conditions. The absorption peak at 1512 cm^−1^ is related to the stretching vibrations of the aromatic skeleton C=C [35], which characterizes both the chemical structure of lignin and of the isocyanate used (TDI). On the other hand, the absorption at 1536 cm^−1^ is attributed to the bending deformations related to the NH group of the formed urethane bond [32]. Thus, this absorption band is absent in the spectra of lignin, while it is found in the spectrum of TDI, respectively, which is a urethane prepolymer containing TDI. In Figure 4d, the band intensities at 1536 and 1512 cm^−1^, respectively, therefore well-represent the ratio between TDI and OL in the three different formulations. The stretching vibration at 1225–1218 cm^−1^ corresponds to the C-N linkage [33,36] in the urethane group, while the band at 1218 cm^−1^ is assigned to S and G ring breathing in lignin [37], with both bands visible in all three formulations and under both crosslinking conditions. An increase of the absorbance at 1225 cm^−1^ can be observed in the formulation PU-1:3, especially in the case of controlled crosslinking (Figure 4c), indicating increased urethane bond formation with higher lignin content and temperature-induced curing of the respective formulation. The presence of an absorption band at around 1067 cm^−1^ indicates the stretching deformation related to the urethane C-O-C soft bond [38]. After crosslinking treatment in the oven, increased absorption at this frequency is observed in the formulations with a higher lignin content (PU-1:3) compared to curing at room temperature, in which the maximum absorption is observed for the formulation with higher isocyanate content (PU-3:1). The fact that in the FTIR spectrum of OL, no absorbance is observed at this frequency, strongly indicates the successful formation of urethane bonds in the formulation PU-1:3 after successive crosslinking at 60 °C. This behavior indicates that isocyanate spontaneously tends to react with the hydroxyl groups of water present in the atmosphere. In the case of temperature-induced crosslinking, the vibrational stretching is greater in the formulation with a greater amount of lignin (PU-1:3), indicating a more intense interaction between isocyanate and hydroxyl groups from lignin.

**Figure 5:**
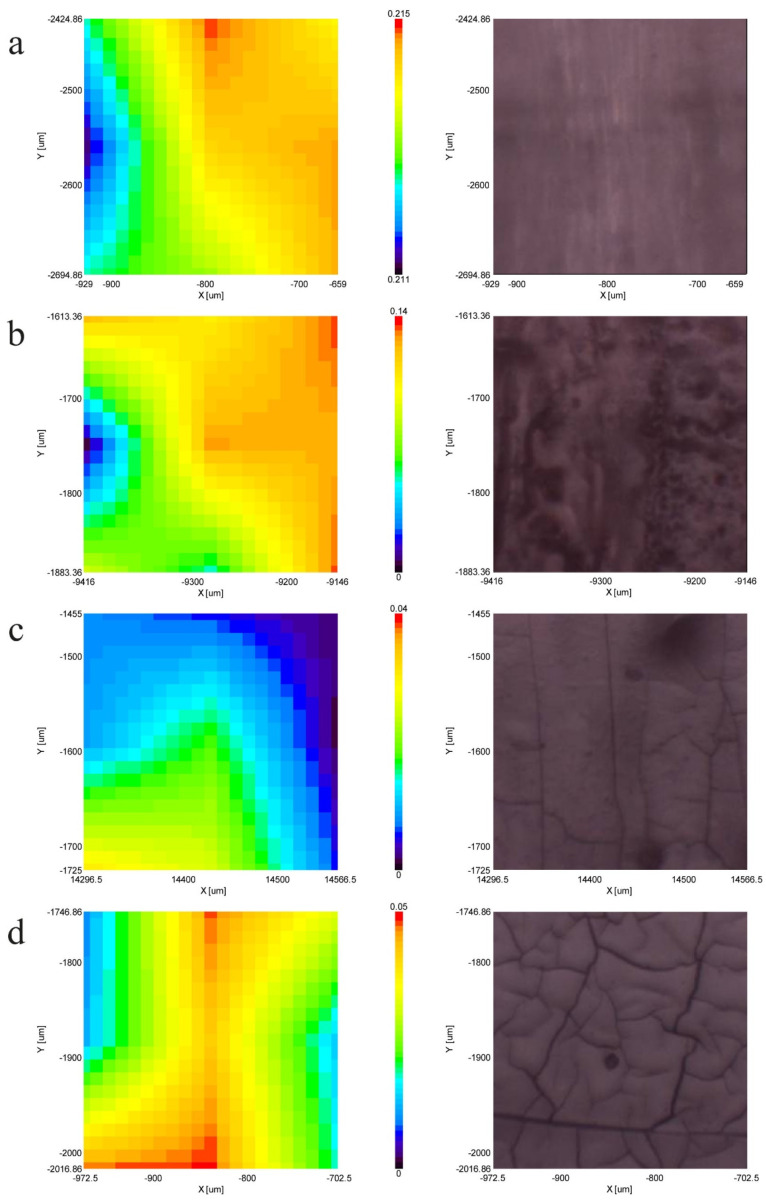
FTIR mapping images based on the absorbance band at 1720 cm^−1^ (left) and respective microscope pictures of the measurement areas (right). (**a**) WPU-R, (**b**) WPU-3:1, (**c**) WPU-1:1 and (**d**) WPU-1:3.

FTIR maps were produced of all three coating formulations applied on wood samples as well as the reference PU coating, respectively, based on the band intensity at 1720 cm^−1^ (Figure 5). The absorbance scale of the reference sample PU-R shows a very narrow range of 0.004 absorbance units, indicating very high homogeneity of the commercial PU coating. Confronting the absorbance scales of the different prepared PU formulations, higher heterogeneity compared to the commercial PU coating is evident. However, a trend to a more homogeneous coating is observed when the lignin content increases. The absorbance of the urethane linkage IR band varies less over the mapped area for the formulations PU-1:1 and PU-1:3. Even if the coatings containing more lignin show superficial defects such as cracks, the rather homogeneous distribution of urethane bonds indicates successful crosslinking of the lignin-based PU coatings and a promising base for the development of bio-based PU coating solutions.

### 3.6. Pyrolysis-GC/MS

**Figure 6:**
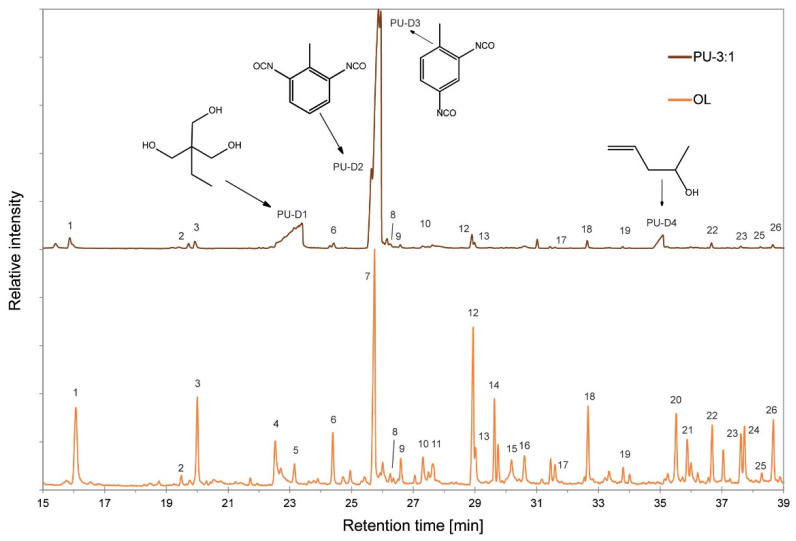
Pyrograms of the Organosolv lignin (OL) used and one of the produced PU sample (PU-3:1).

Figure 6 shows the pyrograms of the lignin sample OL and one PU formulation (PU-3:1) synthesized based on OL. The numbered peaks of the respective pyrolysis products are listed in Table 5. The S/G ratio of the utilized OL, which was isolated from beech wood, is much lower than that calculated for a beech lignin sample isolated using mild acidolysis [39]. However, the fact that the OL sample used here was isolated using a rather harsh Organosolv process that results in degradation of the lignin macromolecule could explain why the S/G ratios of OL differ from values determined for a mildly isolated lignin sample. For the sample PU-3:1, the S/G ratio could not be determined correctly since the peaks of the pyrolysis products of PU overlapped with the respective peaks of major lignin pyrolysis products, and therefore the contribution of the respective compounds to the peak area was not conclusive. In the pyrogram of the PU sample, PU degradation products such as trimethylolpropane (PU-D1), isomers of toluene diisocyanate (PU-D2 and PU-D3) and 4-penten-2-ol (PU-D4) form the major peaks, while lignin pyrolysis products are detected at lower abundancies (Table 5). The strong excess of PU degradation products compared to lignin-derived compounds derives from the ratio of TDI:lignin of 3:1 in the analyzed sample (PU-3:1). Interestingly, the PU degradation products represent the main components of the commercial isoyanate Desmodur^®^L75 used in this study plus the compound PU-D4 deriving from further degradation of diethylenglycol. This degradation pattern cleaving the urethane bonds in the PU network is in concordance with thermal degradation mechanisms of polyurethanes reported by Jiao et al. [40] and indicates a straightforward polymerization reaction without side products’ creation between the commercial isocyanate and the OL used as a polyol in the prepared PU formulations.

## 4. Conclusions

In conclusion, with the increasing lignin content, the color change between uncoated and coated wood increased, tending to red-yellow darker tones. On the other hand, the commercial PU coating barely affected the color change. In addition, WPU-R is transparent, in strong contrast to the prepared lignin-based PU coatings, which made the wood surface opaque. For a higher lignin concentration, solid content, and consequently weight gain, increased as well, whereas WPU-R had a higher SC and weight gain supposedly because of different additives. Coating film profiles appeared more homogenous for the commercial coating than for the prepared formulations, even if an application by brush generally renders the film profile slightly irregular. Contact angles decreased with higher lignin contents and were always lower compared to standard PU coatings at the used lignin concentrations. However, confronting the determined contact angles with the ones observed with virgin wood, an increase in hydrophobicity of the wood surfaces was evident. The lignin concentration also affected the general appearance of the coatings, which presented many cracks for higher lignin contents.

FTIR spectroscopy showed the chemical incorporation of lignin in the structure of the prepared polyurethanes, creating urethane bonds between the applied and the isocyanate and hydroxyl groups of lignin, especially at higher lignin contents. The use of an FTIR microscope for the construction of FTIR maps proved very useful in order to evaluate the homogeneity of the surfaces of PU coating films. Py-GC/MS of the prepared PU formulation further strongly indicated a successful incorporation of lignin in the polyurethane network, since no side-products were detected, and thermal degradation of the PU resulted in the original components of the commercial isocyanate on one side and usual lignin pyrolysis products on the other.

## Figures and Tables

**Table 1:** Formulations of the different coatings and corresponding abbreviations used in the text.

Abbreviation	Formulation
PU-R	Reference sample: commercial polyurethane-based coating
PU-3:1	Lignin-based polyurethane, isocyanate:lignin ratio = 3:1
PU-1:1	Lignin-based polyurethane, isocyanate:lignin ratio = 1:1
PU-1:3	Lignin-based polyurethane, isocyanate:lignin ratio = 1:3
W-R	Reference sample: uncoated beech wood
WPU-R	Reference sample: beech wood coated with PU-R
WPU-3:1	Beech wood coated with PU-3:1
WPU-1:1	Beech wood coated with PU-1:1
WPU-1:3	Beech wood coated with PU-1:3

**Table 2:** Color coordinates based on CIELAB color space of wooden samples before coating application (W-R) and after coating application. ΔE* was calculated using the difference between uncoated (W-R) and coated wood samples. Statistical differences are reported as: *** *p* < 0.001, ** *p* < 0.01 and * *p* < 0.05.

	Before Coating (W-R)			After Coating			
	L*	a*	b*	L*	a*	b*	ΔE*
WPU-R	85.8 ± 3.8	6.4 ± 2.2	19.8 ± 2.0	71.8 ± 2.0 ***	11.3 ± 1.1	31.4 ± 2.6	19.5 ± 4.9 ***
WPU-3:1	85.6 ± 6.7	6.0 ± 2.4	20.3 ± 2.6	37.6 ± 5.3 ***	26.4 ± 2.6 ***	30.0 ± 5.5	54.7 ± 7.6 ***
WPU-1:1	86.2 ± 3.5	5.9 ± 2.0	21.3 ± 2.0	21.0 ± 4.3 ***	21.1 ± 4.2 ***	11.4 ± 5.8 ***	68.7 ± 5.7 ***
WPU-1:3	90.1 ± 2.4	4.0 ± 1.8	19.7 ± 2.4	10.8 ± 2.5 ***	7.8 ± 5.7	0.3 ± 1.0 ***	82.3 ± 3.9 ***
Mean W-R	86.9 ± 7.3	5.6 ± 2.9	20.3 ± 3.0				

**Table 3:** Weight of the samples before and after application of the different coating formulations and respective weight gain, solid content (SC) of the pure formulations (^1^ PU-R, ^2^ PU-3:1, ^3^ PU-1:1, ^4^ PU-1:3). Statistical differences are reported as: *** *p* < 0.001, ** *p* < 0.01 and * *p* < 0.05.

	Weight Gain (%)	Thickness (μm)	SC (%)
WPU-R	5.2 ± 1.7	28.2 ± 20.6	57.1 ^1^
WPU-3:1	0.6 ± 1.7 ***	25.9 ± 7.0	45.1 ^2^
WPU-1:1	0.8 ± 1.6 ***	28.1 ± 7.1	47.1 ^3^
WPU-1:3	0.7 ± 1.3 ***	27.8 ± 9.2	51.0 ^4^

**Table 4:** Contact angle (CA) measured for different prepared PU formulations. Values of CA after waterdrop deposition (CA-T0), CA after 10 s (CA-T10) and CA after 10 min (600 s) after waterdrop deposition (CA-T600) are reported. Statistical differences are reported as: *** *p* < 0.001, ** *p* < 0.01 and * *p* < 0.05.

	CA-T0 (°)	CA-T10 (°)	CA-T600 (°)
W-R	93.9 ± 4.0	76.6 ± 5.9	-
WPU-R	89.5 ± 6.1	89.1 ± 6.2	79.9 ± 6.6
WPU-3:1	82.0 ± 8.2	79.7 ± 7.8	69.1 ± 9.6
WPU-1:1	79.8 ± 8.1	77.3 ± 8.5	54.7 ± 10.7 **
WPU-1:3	79.0 ± 7.7	70.4 ± 5.4 **	-

**Table 5:** Retention times, relative abundances, assignment to guaiacyl-type (G) or syringyl-type (S) aromatic lignin subunits of pyrolysis products and respective S/G ratios of the Organosolv lignin (OL) used and one of the produced PU sample (PU-3:1).

#	Pyrolysis Product	RT (min)	PU-3:1 (%)	OL (%)
1	Guaiacol	16.07	1.3	6.7
2	5-Methylguaiacol	19.48	0.4	0.5
3	Creosol	20.01	0.7	4.6
4	5-Methoxyresinol	22.53	-	2.8
PU-D1	Trimethylolpropane	23.13	15.0	-
5	4-Ethylguaiacol	23.15	-	1.0
6	4-Vinylguaiacol	24.39	0.7	2.6
PU-D2	2.6-Diisocyanate toluene	25.65	9.1	-
7	Syringol	25.75	-	15.1
PU-D3	2,4-Diisocyanate toluene	25.95	63.3	-
8	Dihydroeugenol	26.25	-	0.4
9	Methylsyringol	26.60	0.2	1.1
10	Vanillin	27.31	0.1	1.6
11	Isoeugenol, cis	27.63	-	1.4
12	Vanillic acid	28.94	1.1	8.5
13	Isoeugenol, trans	29.01	0.4	1.6
14	Lauryl chloride	29.63	-	3.4
15	1-Dodecanol	29.75	-	1.6
16	Acetoguaiacone	30.18	-	0.9
17	Guaiacylacetone	31.59	0.1	0.9
18	3,5-Dimethoxyacetophenone	32.67	0.6	3.9
19	Methoxyeugenol	33.80	0.1	0.7
PU-D4	4-Penten-2-ol	35.08	2.7	-
20	Syringaldehyde	35.52	-	3.7
21	Myristyl chloride	35.89	-	2.7
22	Ferulic acid	36.68	0.4	2.8
23	Acetosyringone	37.61	0.1	2.6
24	Myristyl alcohol	37.73	-	2.7
25	Stearic acid	38.29	0.1	0.4
26	Homosyringic acid	38.67	0.3	3.2
	S/G ratio		-	1.0

## Data Availability

The data presented in this study are available on request from the corresponding author mroma@unitus.it.
